# Rate of Primary Refractory Disease in B and T-Cell Non-Hodgkin’s Lymphoma: Correlation with Long-Term Survival

**DOI:** 10.1371/journal.pone.0106745

**Published:** 2014-09-25

**Authors:** Corrado Tarella, Angela Gueli, Federica Delaini, Andrea Rossi, Anna Maria Barbui, Giuseppe Gritti, Cristina Boschini, Daniele Caracciolo, Riccardo Bruna, Marco Ruella, Daniela Gottardi, Roberto Passera, Alessandro Rambaldi

**Affiliations:** 1 Department of Biotechnology and Life Sciences, University of Torino, Torino, Italy; 2 Hematology and Cell Therapy Division, Mauriziano Hospital, Torino, Italy; 3 Hematology and Bone Marrow Transplant Units, A. O. Papa Giovanni XXIII, Bergamo, Italy; 4 Division of Hematology I, A. O. Città della Salute, Torino, Italy; 5 Division of Nuclear Medicine, University of Torino, Torino, Italy; European Institute of Oncology, Italy

## Abstract

**Background:**

Primary refractory disease is a main challenge in the management of non-Hodgkin’s Lymphoma (NHL). This survey was performed to define the rate of refractory disease to first-line therapy in B and T-cell NHL subtypes and the long-term survival of primary refractory compared to primary responsive patients.

**Methods:**

Medical records were reviewed of 3,106 patients who had undergone primary treatment for NHL between 1982 and 2012, at the Hematology Centers of Torino and Bergamo, Italy. Primary treatment included CHOP or CHOP-like regimens (63.2%), intensive therapy with autograft (16.9%), or other therapies (19.9%). Among B-cell NHL, 1,356 (47.8%) received first-line chemotherapy with rituximab. Refractory disease was defined as stable/progressive disease, or transient response with disease progression within six months.

**Results:**

Overall, 690 (22.2%) patients showed primary refractory disease, with a higher incidence amongst T-cell compared to B-cell NHL (41.9% vs. 20.5%, respectively, p<0.001). Several other clinico-pathological factors at presentation were variably associated with refractory disease, including histological aggressive disease, unfavorable clinical presentation, Bone Marrow involvement, low lymphocyte/monocyte ration and male gender. Amongst B-cell NHL, the addition of rituximab was associated with a marked reduction of refractory disease (13.6% vs. 26.7% for non-supplemented chemotherapy, *p*<0.001). Overall, primary responsive patients had a median survival of 19.8 years, compared to 1.3 yr. for refractory patients. A prolonged survival was consistently observed in all primary responsive patients regardless of the histology. The long life expectancy of primary responsive patients was documented in both series managed before and after 2.000. Response to first line therapy resulted by far the most predictive factor for long-term outcome (HR for primary refractory disease: 16.52, *p*<0.001).

**Conclusion:**

Chemosensitivity to primary treatment is crucial for the long-term survival in NHL. This supports the necessity of studies aimed to early identify refractory disease and to develop different treatment strategies for responsive and refractory patients.

## Introduction

Despite improvements in the efficacy of the available treatments, there is a variable proportion of non-Hodgkin’s Lymphoma (NHL) patients displaying very poor or transient response to primary treatment. [1]–[3] These patients have primary refractory disease. At present, primary refractoriness remains a challenge in the management of malignant lymphoma. [4], [5] In fact, several studies are investigating molecular markers that may be associated with refractory disease. [6]–[11] These markers might allow for early diagnosis, as well as the identification of novel therapeutic targets. [12]–[14] Moreover, alternative treatment options are sought in order to improve the usually poor outcome for refractory lymphoma patients. [15]–[17].

Although response to primary treatment has relevant clinical implications, there are several open issues regarding primary refractory disease. In particular, it has to be determined: (i) the real proportion of refractory patients amongst the various lymphoma subgroups; (ii) the influence of the presently available treatments on the rate of refractory disease; (iii) the actual long-term survival of primary responsive compared to primary refractory patients.

To address these issues, we performed a long-term, retrospective survey on 3,106 NHL patients that had been managed over the last three decades. Aims of the study were to define the rate of responsiveness among patients requiring primary systemic treatment for their newly diagnosed malignant lymphoma, and to outline the impact of the response to primary treatment on the overall survival.

## Methods

### Data sources, patient population, and clinical procedures

The retrospective review was performed on newly diagnosed, NHL patients, admitted and managed at the University Hematology of Torino (S. Giovanni Battista and Mauriziano Hospitals) and at the Hematology Division of Ospedali Riuniti of Bergamo, Italy, during the last three decades (1982–2012). An electronic database has been used since early 1980 s at the Hematology Division of Bergamo. [18] A similar database has been used since 2000 at the University Department of Hematology in Torino. [19] Based on these registries, data have been collected of 3,393 NHL patients requiring systemic therapy. The retrospective analysis was approved by our local Ethical Committee (Ospedale Infantile Regina Margherita [O.I.R.M.]/Sant’Anna - Ordine Mauriziano, Torino) (prot.No. 100355/A210). The Ethical Committee did not require a written informed consent form for the patients included in the analysis. However, patient information was anonymized in the recorded files.

All patients underwent common diagnostic procedures, in order to define the type of lymphoma and disease extension, and then to evaluate response to treatment. A total of 3,393 patients entered in our data base, however information on response to primary treatment was lacking for 78 (2.3%) of them and overall3,315 could be considered for the analysis. In addition, 209 patients (6.3%), with a median age of 69 (range, 20–98), died early (within 8 months) during or before any treatment was given, and could not be properly evaluated for response. Causes of early death were: i. early, treatment related toxicities (n = 163, 4.9% of the whole series), including cardiac, hepatic, renal, pulmonary or infectious causes; ii. other cancers (n = 8, 0.2%); iii. unknown reasons (n = 17, 0.5%); iv. probable but not formally proven lymphoma progression (n = 21, 0.6%). Thus, we were able to complete an extensive analysis on response to primary systemic treatment on 3,106 patients.

All patients received chemotherapy, with or without rituximab, depending on the time of diagnosis in relationship to the time of rituximab availability in the clinical setting. [20], [21] Overall, patients were managed with three main treatment strategies: (i) CHOP or CHOP-like schedules, including MACOP-B, VACOP, ACOP, CNOP, COMP, and CHOEP; [22], [23] (ii) high-dose sequential program with autograft (HDS regimen); [19] (iii) a miscellaneous group of other therapies, mostly including the different schedules that are variously employed for low-grade lymphoma, such as single-agents, Chlorambucil, Fludarabine, Cladribine, Mechlorethamine, Cyclophosphamide, Bendamustine, or combination schedules, such as CVP, FND, DHAP, MINE, or intensive programs (BFM or Magrath-schedule) for Burkitt’s or Burkitt-like lymphoma. [1], [4], [24]–[26].

### Study outcomes

The main objectives of the study were: (i) to define the rate of primary refractory disease; (ii) to evaluate the clinical and therapeutic factors associated with refractory disease; and (iii) to investigate the overall survival (OS) of refractory vs. responsive patients, according to Cheson criteria. [27] Refractory disease was defined as:

- stable or progressive disease (fully refractory), following front-line therapy, either completed or discontinued in order to shift to an intensified salvage program;
- transient response with disease progression within six months (early progression), following first-line chemotherapy.

### Statistical analysis

Patient characteristics were tested using the Fisher’s exact test for categorical variables and the Mann-Whitney test for continuous ones. For univariate survival analyses, the OS curves were first estimated by the Kaplan-Meier method, and compared using the log-rank test, then the Cox proportional hazards model was used to compare risk factors by the Wald test [28], [29]. Comparison included the following parameters: gender, age at diagnosis (>60 vs. ≤60 yrs), histological subtypes, IPI score (3–5 vs. 0–2), bone marrow (BM) involvement, rituximab administration, lymphocyte to monocyte ratio at diagnosis (≤2.6 vs.>2.6) [30], presence and type of primary refractory disease, and the administration of HDS front-line. The multivariate Cox model was also used to assess the effects of these risk factors on OS; all the above-mentioned covariates, except gender, were treated as time-dependent variables, similar to the univariate ones. At last, the same predictors were used as independent variables in different univariate and multivariate binary logistic regression models, in order to identify the possible risk factors for the status of refractoriness (dependent variable); these results are presented as OR and 95% CIs. The diagnostic performances of these models are expressed as accuracy, sensitivity, specificity, and positive/negative predictive values. All reported *p*-values were two-sided, at the conventional 5% significance level. Data were analyzed as of October 2013, using IBM SPSS 21.0.0 and R 3.0.0. At the time of analysis, there were 1,864 (60%) patients known to be alive: 1,508 (80.9%) of them had been seen in the clinic or had been contacted by phone at least once over the last 18 mos., whereas 356 (19.1%) patients had been followed for a median of 6.5 years (range 0.5–29 years), afterwards they discontinued the follow up.

## Results

The main clinical characteristics of the whole series of 3,315 patients that were analyzed are summarized in **Table 1**. There were no significant differences in the distribution of the main clinical parameters between the Bergamo and Torino Centers. All patients who had undergone primary systemic treatment were considered eligible for the study, including patients with very advanced age. Indeed, there were 148 patients (4.5%) over 80 years old. Among initial 3,315 patients, 209 patients had an early death, mainly due to toxicity (see Patients and Methods and Table 1). Thus, the rate of response to first line therapy could be properly determined in 3,106 patients.

**Table 1:** Characteristics of the Study Cohort.

Characteristic	Patients *(N = 3,315)* 1
Age – *yr* median (range)	59 (15–98)
Male sex – *no. (%)*	1,808 (54.5)
Histology - *no. (%)*	
• B cell	3,066(92.5)
• T cell	249(7.5)
B-cell subtypes^2^– *no. (%)*	
• DLB-CL	1,694 (55.3)
• FL	607 (19.8)
• MCL	196 (6.4)
• Miscellaneous	569(18.5)
Histological grade^3^ - *no. (%)* *	
• low grade	992 (29.9)
• intermediate/high grade	2,322 (70.1)
Ann Arbor Stage - *no. (%)* **	
• I–II	1,070 (33)
• III–IV	2,172 (67)
BM involvement - *no. (%)* ***	
• NO	1,818 (65.2)
• YES	971 (34.8)
LDH serum level - *no. (%)* **^†^**	
• normal	1,657 (58.7)
• high	1,167 (41.3)
IPI score^4^ - *no. (%)* **^††^**	
• 0–2	1,448 (58)
• 3–5	1049 (42)
LM ratio^5^ - *no. (%)* **^‡^**	
• >2.6	1,281 (59.3)
• ≤2.6	879 (40.7)
Chemotherapy schedule^6^ - *no. (%)* **^†††^**	
• CHOP or CHOP-like	2,089 (64)
• HDT and autograft	529 (16.2)
• other schemes	648 (19.8)
Rituximab addition among B-cell subtypes - *no. (%)* **^§^**	
• NO	1,595(53.2)
• YES	1,405(46.8)
Referral Center	
• Torino	840(25.3)
• Bergamo	2,475(74.7)

1 Among 3,315 assessable patients in our data base, 209 died early, due to early toxic death (n = 163, 4.9%), other cancers (n = 8, 0.2%), unknown reasons (n = 17), probable but not formally proven lymphoma progression (n = 21); overall, 3,106 could be properly evaluated for response to therapy;^2^B-cell lymphoma was classified into four groups, i.e.: Diffuse Large B-Cell Lymphoma (DLB-CL), Follicular Lymphoma (FL), Mantle-Cell Lymphoma (MCL), and miscellaneous histologies, including marginal-zone (MZL), small lymphocytic (SL), and Burkitt’s and lymphoblastic lymphoma; ^3^low-grade lymphoma included FL-MZL-SL-low-grade T-cell lymphoma, all remaining subtypes, i.e. MCL, DLCL, transformed-FL, high-grade peripheral T-cell NHL, other aggressive histotypes (Burkitt’s and Burkitt-like NHL, Lymphoblastic lymphoma) were included among intermediate/high-grade histologies; ^4^IPI: International Prognostic Index: assessed in all diffuse large-cell lymphoma, and low-grade lymphoma; ^5^the Lymphocyte to Monocyte (LM) ratio was assessed at diagnosis by automatic blood count; ^6^Chemotherapy was delivered to all patients, according to various schedules, as detailed in the text.

missing values: * = 1; ** = 73 (2.2%); *** = 526 (15.9%); ^†^ = 491 (14.8%); ^††^ (including cases where IPI was NA) = 818 (24.7%); ^‡^ = 1,155 (34.8%); ^†††^ = 49 (1.5%); ^§^ = 66 (2.1%).

### Raw incidence of refractory disease

As reported in **Table 2**, 690 (22.2%) out of 3,106 assessable patients showed refractory disease, with similar frequencies in Bergamo and Torino Centers. A markedly higher raw incidence of refractory disease was observed amongst T-cell compared to B-cell NHL (41.9% vs. 20.5%, respectively, *p*<0.001). In addition, aggressive disease, defined in terms of either histology or clinical prognostic presentation, was associated with significantly increased incidence of refractory disease. Female patients were slightly, but significantly, more responsive than male patients. By contrast, the overall frequency of refractory patients was not significantly influenced by age (23.1%, above 60 years, vs. 21.5%, 60 or younger). Lastly, an intensive primary therapy with autograft was associated with a significantly higher response compared to conventional chemotherapy regimens (overall refractory disease: 19% vs. 22.7%, respectively, *p* = 0.012).

**Table 2:** Raw Incidence of Primary Responsive vs Primary Refractory Disease, According to Main Clinical and Therapeutic Factors, Among 3,106 Patients Evaluable for Response to First Line Therapy.

Parameter	Responsive *n = (%)*	Fully refractory *n = (%)*	Early progression *n = (%)*	*p = *
all patients	2,416 (77.8)	386 (12.4)	304 (9.8)	–
Center				
• Bergamo	1,780 (78.3)	274 (12.0)	220 (9.7)	0.509
• Torino	636 (76.4)	112 (13.5)	84 (10.1)	
Gender				
• Female	1,138 (80.9)	148 (10.5)	121 (8.6)	0.001
• Male	1,278 (75.2)	238 (14.0)	183 (10.8)	
Age				
• ≤60 yrs.	1,335 (78.5)	204 (12.0)	161 (9.5)	0.547
• >60 yrs.	1,081 (76.9)	182 (12.9)	143 (10.2)	
Main histology I.				
• B-cell	2,272 (79.5)	317 (11.1)	269 (9.4)	<0.001
• T-cell	144 (58.1)	69 (27.8)	35 (14.1)	
Main histology II.				
• int/high-grade	1,605 (75.0)	310 (14.5)	224 (10.5)	<0.001
• low-grade	810 (83.9)	76 (7.9)	80 (8.3)	
Ann Arbor Stage				
• I–II	900 (87.3)	69 (6.7)	62 (6.0)	<0.001
• III–IV	1,485 (73.3)	302 (14.9)	239 (11.8)	
IPI				
• 0–2	1,241 (87.4)	92 (6.5)	87 (6.1)	<0.001
• 3–5	669 (67.8)	186 (18.9)	131 (13.3)	
LM ratio				
• >2.6	1,023 (83.7)	101 (8.3)	98 (8.0)	0.311
• ≤2.6	625 (77.5)	115 (14.3)	66 (8.2)	
BM involvement				
• NO	1,434 (82.5)	168 (9.7)	135 (7.8)	0.001
• YES	673 (73.6)	133 (14.6)	108 (11.8)	
Chemotherapy schedule				
• Conventional chemotherapy	1,961 (77.3)	331 (13.0)	245 (9.7)	0.012
• HDT and autograft	425 (81.0)	44 (8.4)	56 (10.7)	

**Figure 1:**
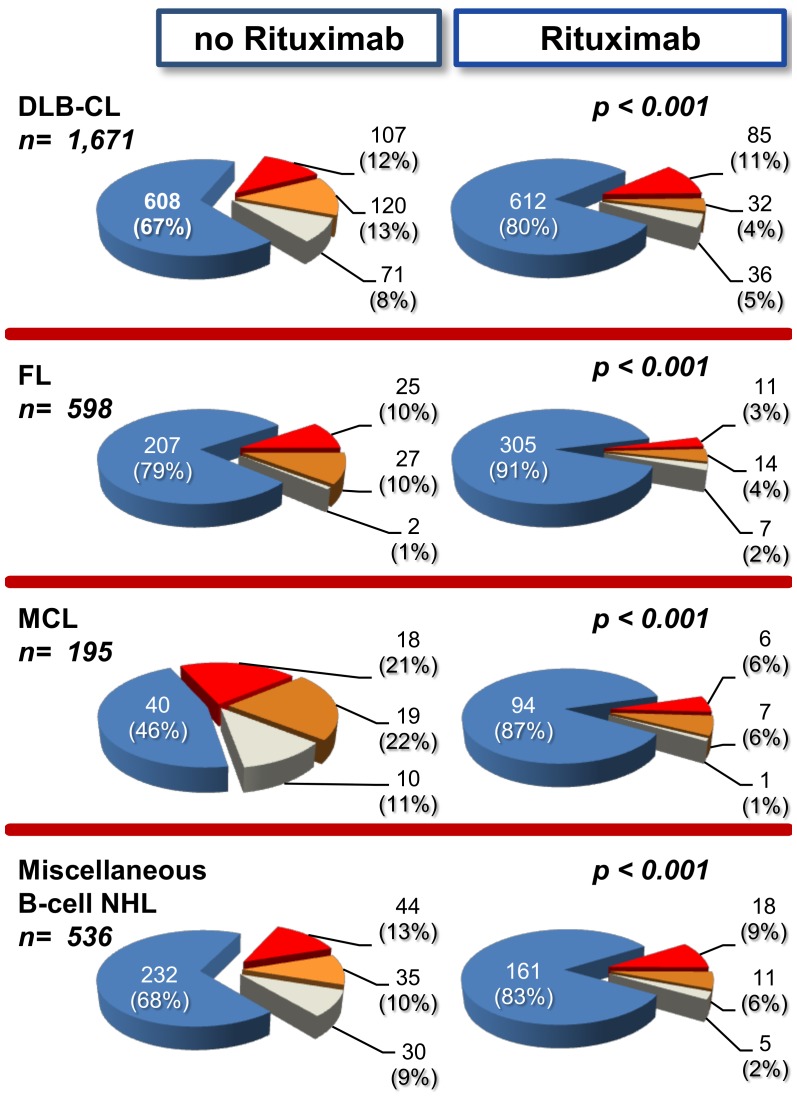
Raw Incidence of Primary Responsive vs Primary Refractory Patients Among Main B-Cell Lymphoma Subtypes, According to Rituximab Administration. Blue = responsive, red = fully refractory, brown = early progression, grey = early death. No Rituximab = Chemotherapy without Rituximab; Rituximab = chemotherapy supplemented with Rituximab. DLB-CL: Diffuse Large B-Cell Lymphoma; FL: Follicular Lymphoma; MCL: Mantle-Cell Lymphoma; Miscellaneous B-cell NHL: marginal-zone, small lymphocytic, Burkitt’s and lymphoblastic lymphoma. Data on Rituximab administration were lacking on 21 out of 2,858 B-cell lymphoma patients. *p values* were calculated for responsive/refractory ratio in No Rituximab compared to Rituximab.

Among B-cell NHL, patients receiving rituximab-supplemented chemotherapy (n = 1,356) had a marked reduction of overall refractory disease (13.6%, including 8.8% of fully refractory and 4.7% of early progression) compared to patients receiving non-supplemented chemotherapy (n = 1,481), where refractory disease resulted of 26.7%, including 8.8% of fully refractory and 4.7% of early progression (*p*<0.001). As shown in **Figure 1**, the overall rate of primary refractory disease was significantly reduced in all B-cell subtypes when chemotherapy was given with rituximab addition compared to non-supplemented chemotherapy.

When assayed in multivariate binary logistic regression analysis, several clinical and therapeutic factors maintained their independent association with either fully refractory disease or early progression or both, as detailed in **Table 3**.

**Table 3:** Multivariate Binary Logistic Regression Analyses on Factors Associated with Refractory Disease.

Parameters associated with:	All evaluable patients (*n = 3,106)*			B-cell subtype (*n = 2,858*)		
	O R^1^	95% C.I.	*p = *	O R^1^	95% C.I	*p = *
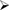 ***Fully refractory*** ** ***vs responsive disease:***						
T-cell vs. B-cell histology	1.86	1.06–3.28	0.030	NA	NA	NA
Int/high vs. low grade histology	1.74	1.09–2.78	0.019	1.71	1.07–2.72	0.024
IPI score 3–5 vs. 0–2	4.18	2.94–5.96	<0.001	3.74	2.57–5.44	<0.001
Rituximab adm. Yes vs. No	0.43	0.30–0.62	<0.001	0.43	0.30–0.62	<0.001
HDT &autograft Yes vs. No	0.41	0.24–0.72	0.002	0.41	0.22–0.76	0.005
Gender F vs. M	0.74	0.53–1.04	0.085	0.69	0.48–0.99	0.044
LM ratio ≤2.6 vs>2.6	1.57	1.11–2.23	0.011	1.79	1.24–2.59	0.002
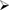 ***early progression vs responsive disease:***						
Int/high vs. low grade histology	1.64	0.92–2.90	0.092	1.68	0.94–3.03	0.082
IPI score 3–5 vs. 0–2	2.24	1.36–3.70	0.002	1.99	1.15–3.43	0.014
Rituximab adm. Yes vs. No	0.15	0.09–0.24	<0.001	1.16	0.10–0.27	<0.001
BM involvement Yes vs. No	2.26	1.35–3.80	0.002	2.79	1.60–4.87	<0.001
LM ratio ≤2.6 vs>2.6	1.47	0.93–2.31	0.099	1.30	0.79–2.15	0.300
HDT &autograft Yes vs. No	0.50	0.24–1.05	0.066	0.46	0.19–1.09	0.078
Gender F vs. M	0.71	0.46–1.11	0.135	0.58	0.35–0.94	0.028

### Long-term outcome

**Figure 2:**
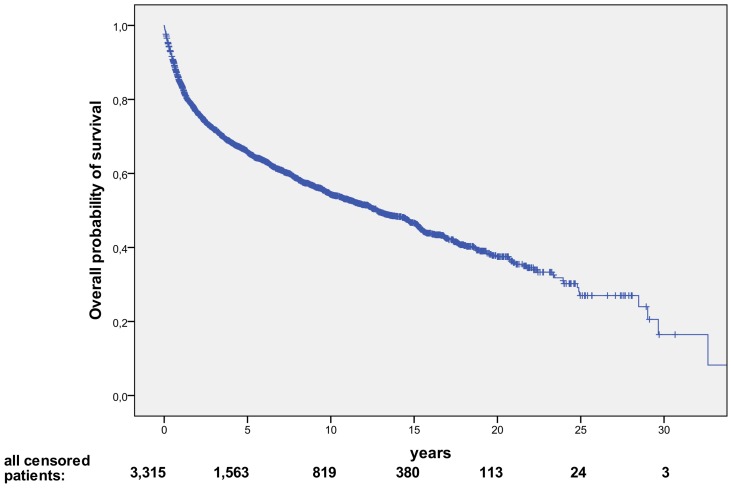
Overall Survival of 3,315 Non-Hodgkin’s Lymphoma (NHL) Patients following first line therapy. At a median follow-up of 7.3 years., the median survival for the entire cohort was of 12.7 yrs. (range: 0–32.6yrs).

The overall survival curve for the whole series of 3,315 patients is reported in Figure 2.

The long-term outcome has been then evaluated in the 3,106 patients assessable for response to primary treatment. As of October 2013, 1,864 (60.0%) were known to be alive, and at a median follow-up of 7.5 yrs, the 5, 10 and 15 yr. survival projections were 69.9%, 57.9% and 49.2%, respectively, with a median survival of 14.6 yrs. Patients with B-cell lymphoma had a much longer overall survival than those with T-cell subtypes, with median survival of 15.0 and 6.4 yrs, respectively. Among B-cell subtypes, the median survival was 25.0, 15.0, and 5.7 yrs, for FL, DLB-CL, and MCL, respectively (P<0.001). Moreover, B-cell NHL had a marked survival improvement since the addition of rituximab to chemotherapy, with a median survival of 11.4 yrs for patients treated without rituximab, whereas the median survival has not yet been reached for patients treated with chemotherapy plus rituximab. For these latter patients, the 5, 10 and 15 yr survival projections are 79.3%, 70.0% and 63.0%, respectively (data not shown).

**Figure 3:**
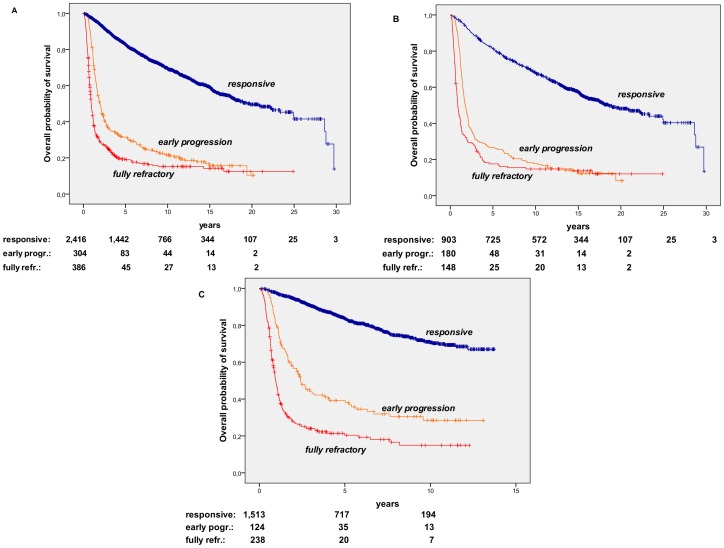
Overall Survival in Non-Hodgkin’s Lymphoma (NHL) Patients according to response to primary therapy. A. Overall survival (OS) of 3,106 NHL patients diagnosed and managed during the period 1982–2012. The OS projections are of 82.8%, 69.5%, and 59.2%, for responsive patients, 31.5%, 21.6%, and 15.7% for early progression, 18.9%, 15.2%, and 14.2% for fully refractory patients, at 5, 10, and 15 years, respectively (responsive vs. refractory: p<0.001). Median follow-up for the whole series is 7.5 yrs (range, 0.6–31.2). B. OS of 1,231 NHL patients diagnosed and managed up to 1,999. The OS projections are of 81.4%, 68.1%, and 57.6%, for responsive patients, 26.7%, 17.7%, and 12.4% for early progression, 17.6%, 14.8%, and 13.8% for fully refractory patients, at 5, 10, and 15 years, respectively (responsive vs. refractory: p<0.001). Median follow-up for the whole series is 16 yrs (range, 0.6–31.2) C. OS of 1,875 NHL patients diagnosed and managed since 2,000. The OS projections are of 83.8% and 70.7%, for responsive patients, 39.1% and 28.4%, for early progression, 20.4% and 14.9%, for fully refractory patients, at 5 and 10 years, respectively (responsive vs. refractory: p<0.001). Median follow-up for the whole series is 5.1 yrs (range, 0.6–13.7).

Overall, primary responsive patients had a very prolonged life expectancy, with a median survival of 19.8 yrs., whereas patients with primary refractory disease had a markedly short survival, as shown in **Figure 3A**. Indeed, fully refractory patients had an even shorter median survival of 10.8 mos., compared to 1.9 yrs for early progression patients. The favorable outcome of primary responsive compared to primary refractory patients was reliably recorded both among patients registered up to 1,999 (**Figure 3B**) and those included after 2,000 (**Figure 3C**). A slight though significant improvement in life expectancy was observed by comparing patients registered in the period up to 1,999 vs. those diagnosed and treated after 2,000, both among primary responsive patients (median survival 18,2 yrs vs. not reached, respectively) and early progression patients (median survival 1.75 yrs vs. 2.4 yrs., respectively), whereas no significant differences were seen among fully refractory patients (median survivals of 0.86 and 0.95 yr, prior and after 2,000, respectively).

**Figure 4:**
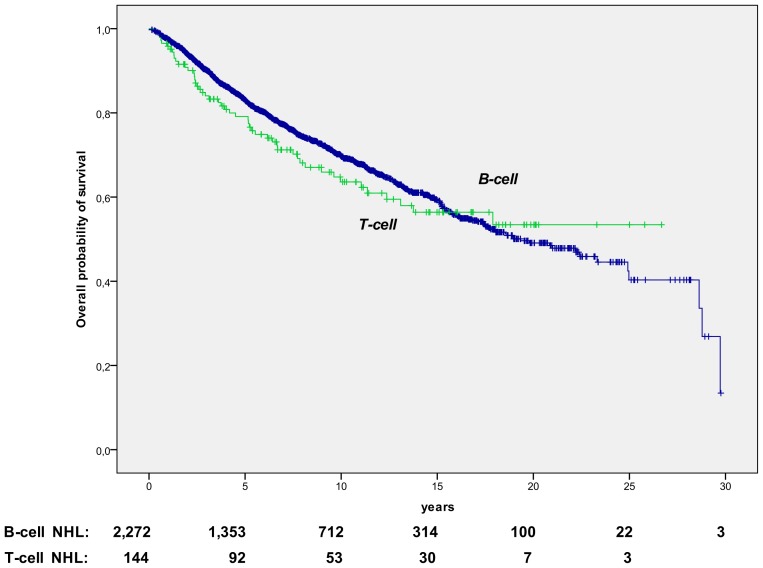
OS in responsive lymphoma, according to T-cell vs. B-cell subtype. The OS projections are of 78.9%, 62.5%, and 55.2%, for T-cell lymphoma patients (median follow up: 10.1 yrs, range, 0.9–26.7), and 83.1%, 69.9%, and 59.5%, for B-cell lymphoma patients (median follow up: 7.5 yrs, range, 0.6–31.2), at 5, 10, and 15 years, respectively (p = 0.326).

The very prolonged survival of responsive patients was equally observed in B-cell and T-cell subtypes (median survivals: 19.3 yrs and not reached, for B-cell and T-cell, respectively), as shown in **Figure 4**.

**Figure 5:**
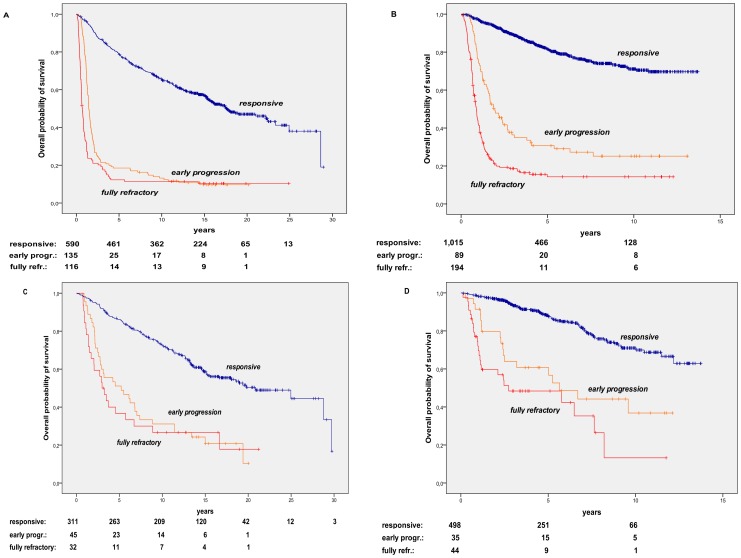
OS in Low-grade and Intermediate/High-grade NHL subtypes according to response to primary therapy. A. OS of 842 Intermediate/High-grade NHL patients diagnosed and managed up to 1,999. The OS projections are of 78.9%, 65.6%, and 57.2%, for responsive patients, 18.5%, 13.2%, and 9.7% for early progression, 12.3%, 11.4%, and 10.4% for fully refractory patients, at 5, 10, and 15 years, respectively. Median follow-up for the whole series is 15.9 yrs (range, 0.6–28.9) B. OS of 1,301 Intermediate/High-grade NHL patients diagnosed and managed since 2,000. The OS projections are of 81.6% and 71.1%, for responsive patients, 30.7% and 25.2% for early progression, 14.3% and 14.3% for fully refractory patients, at 5 and 10 years, respectively. Median follow-up for the whole series is 5.0 yrs (range, 0.6–13.7) C. OS of 388 Low-grade NHL patients diagnosed and managed up to 1,999. The OS projections are of 78.0%, 64.0%, and 51.8%, for responsive patients, 51.1%, 31.1%, and 20.8% for early progression, 36.7%, 26.7%, and 26.7% for fully refractory patients, at 5, 10, and 15 years, respectively. Median follow-up for the whole series is 16.3 yrs (range, 1.7–31.2) D. OS of 574 Low-grade NHL patients diagnosed and managed since 2,000. The OS projections are of 83.5% and 64.2%, for responsive patients, 60.7% and 36.8% for early progression, 48.4% and 13.2% for fully refractory patients, at 5 and 10 years, respectively. Median follow-up for the whole series is 5.1 yrs (range, 0.5–13.7). (responsive vs. refractory: p<0.001, in all series).

The marked difference in survival between primary responsive and primary refractory patients was consistently observed in all subtypes, with distinct OS curves according to histology. **Figures 5** shows the survival curves of the two most representative main histological subgroups, i.e. intermediate/high and low-grade lymphoma (see Table 1 for details), according to the two main periods of patient registration. As shown in Figure 5 A and B, primary responsive intermediate/high grade lymphoma patients had a very prolonged survival, with median survival of 17.7 yrs and not reached, for the series up to 1,999 and since 2,000, respectively. The outcome was very poor for primary refractory patients, with median survival of 1.5 and 2.0 for early progression patients and 0.7 and 0.9 yrs for fully refractory patients, in the up to 1,999 and since 2,000 series, respectively. Figure 5 C and D show the long-term outcome of low-grade lymphoma patients: again, primary responsive patients had a prolonged survival, both in the series registered up to 1,999 and since 2,000, with median survival of 20.8 yrs and not reached, respectively. Among refractory patients, median survival was of was 5.2 and 5.6 for early progression patients and 3.0 and 2.7 yrs for fully refractory patients, in the up to 1,999 and since 2,000 series, respectively.

**Table 4** specifies the main parameters that have independent prognostic value on overall survival. Primary refractoriness was the strongest risk factor, with a higher risk for fully refractory disease compared to early progression. The multivariate Cox proportional hazard regression analysis on factors affecting the OS was also performed on the three main B-cell subgroups, DLB-CL, FL, and MCL. In all subgroups, the high predictive value of primary refractory disease was confirmed (HR 15.2, 13.7, and 21.5, for DLB-CL, FL, and MCL, respectively).

**Table 4:** Multivariate Cox Proportional Hazard Regression Analysis on Factors Affecting the Overall Survival.

Parameter associated with survival*	All evaluable patients *n = 3,106*			B-cell subtype *n = 2,858*		
	H R^2^	95% C.I.	*p = *	H R^2^	95% C.I	*p = *
Age>60 yrs	1.66	1.27–2.18	<0.001	1.76	1.31–2.35	0.001
high vs. low grade histology	2.45	1.76–3.42	<0.001	2.46	1.76–3.44	<0.001
IPI score 3–5 vs. 0–2	1.57	1.28–1.92	<0.001	1.55	1.25–1.91	<0.001
Rituximab adm. Yes vs. No	0.60	0.49–0.73	<0.001	0.60	0.49–0.74	<0.001
Primary Refr.						
• *fully refr*	26.73	18.10–39.47	<0.001	27.03	17.89–40.84	<0.001
• *Early progr*	10.22	7.20–14.50	<0.001	10.92	7.55–15.81	<0.001

*treated as time-dependent variables.

## Discussion and Conclusions

The availability of databases containing large volumes of data on lymphoma patients, managed over the last three decades, allowed us to investigate the role of response to primary treatment on long-term survival. [18], [19], [31] A variable proportion of patients refractory to primary treatment were observed in all lymphoma subgroups, with the highest frequency amongst T-cell subtypes. Amongst B-cell lymphoma, a marked reduction in the frequency of primary refractory disease occurred since the introduction of rituximab to chemotherapy. Overall, response to primary treatment resulted as the key factor for long-term outcome, with a median overall survival of 19.8 years for primary responsive patients, compared to 1.3 years for primary refractory patients.

The series included all evaluable, adult patients that were managed at the Hematology Centers of Torino and Bergamo over the last three decades. Our analysis was performed on an unselected patient population, including all patients older than 14 years, requiring systemic treatment for lymphoma. These patients represent the common lymphoma population that is managed with systemic chemotherapy or chemo-immunotherapy in a hematology Center. [32] B-cell lymphoma was, by far, the most frequent histological form. In particular, there was a marked prevalence in the DLB-CL subtype, followed by FL. Thus, the present analysis reflects the common situations that a hemato-oncologist faces whenever a patient needs treatment for his newly diagnosed lymphoma.

Patients showing stable or progressive disease following primary treatment, or with transient response soon followed by disease progression within six months since therapy completion, are usually classified as primary refractory patients. [4], [5], [13], [27], [33] All lymphoma subtypes showed the presence of a variable proportion of refractory patients, indicating that chemotherapy resistance is a feature that can occur in both aggressive and indolent lymphoma. The highest raw incidence of primary refractory disease was found in the T-cell lymphoma subgroup, with a percentage of refractory patients as high as 41.9%, confirming that the main problem in the management of T-cell lymphoma remains the reduced CR/PR achievement following induction therapy. [2], [34], [35] Indeed, no significant differences were observed between T-cell and B-cell subtypes in the survival curves when the analysis was restricted to responsive patients. Other clinic-pathological variables that were associated with either fully refractory disease or early progression or both were intermediate/high-grade histology, advanced disease presentation, male gender, BM involvement and low lymphocyte/monocyte ratio. Interestingly, advanced age over 60 years was not associated with therapy-resistance. Thus, factors other than disease refractoriness are likely to be responsible for the poorer outcome of elderly lymphoma patients compared to patients younger than 60 years.

Among B-cell types, the addition of rituximab brought a marked fall of refractory disease. This effect was consistently observed in each histological subtype. Since the introduction of rituximab in clinical practice, the chemotherapeutic programs have also been modified, particularly in FL and MCL. [3], [10], [24], [25] Thus the reduction of refractory disease might be ascribed to both the high anti-lymphoma activity of rituximab and to chemotherapy schemes with improved therapeutic efficacy. However, the treatment schedule for aggressive lymphoma has not changed remarkably over the last 20 years, as it remains largely based on the CHOP or CHOP-like schedules.**^21–23^** Thus, at least in the most frequent DLB-CL subtype, rituximab is mainly responsible for the reduction of refractory disease, falling from 27.2% to 16.0%, confirming that rituximab front-line is crucial and may overcome some tumor-associated drug-resistance. [36], [37].

The front-line use of intensive chemotherapy with autograft was associated with a significant reduction of refractory disease. However, this had no significant impact on the overall survival when front-line autograft was evaluated in the multivariate Cox analysis. The early and late fatal toxicities may have offset the increased anti-tumor efficacy of autograft-based front-line therapy. [38] An intensified rescue program with either autologous or allogeneic transplantation should be early considered in those patients showing poor response to standard chemo- or chemo-immunotherapy. At present, there are inadequate data to define which might be the optimal choice, i.e. autologous vs. allogeneic transplantion, for patients with chemorefractory disease. Meanwhile, studies are needed in order to develop novel and effective treatment strategies with reduced toxicity to be employed front-line in those patients at high risk of primary refractory disease.

The prolonged survival of primary responsive patients is an impressive, and somewhat unexpected, finding. Both the analysis of a large patient population and the long-term follow-up have allowed the first documentation that primary responsive lymphoma patients have a global median survival around 19 years. The median survivals of 25 yrs. for responsive FL and 15 yrs. for responsive DLB-CL are definitely unanticipated. The long-term survival is expected to further improve for responsive patients receiving first-line therapy with rituximab. In fact, patients managed since 2,000 show survival projections longer compared to the cohort treated in the period up to 1,999. The survival improvements might be ascribed either to the use of rituximab or to the effective salvage treatments developed in the last decade, or, possibly, to both of these factors. [1], [4], [12], [26], [33] Nevertheless, the analysis demonstrates that primary responsive patients have a very favorable outcome. Currently, patients presenting with advanced-stage B-cell lymphoma have a life expectancy longer than 10 years if they are responsive to their rituximab-containing, first-line chemotherapy.

Since 2,000 when rituximab was introduced in the clinical practice, survival improvements have also been observed for refractory patients, particularly those with initial, though transient, response. The current practice is to shift early to intensive treatments, including autologous or allogeneic transplantation, as soon as therapy resistance is clinically identified. [4], [5], [15], [17], [19], [26], [39] These measures are likely responsible for the improved survival of refractory patients treated in the last 13 years. Nevertheless, the long-term outcome of primary refractory patients remains poor, particularly those with intermediate/high grade histology and fully refractory disease. In addition, the Cox multivariate analysis on the whole series of 3,106 patients indicates refractory disease as the most predictive factor for the long-term outcome, with the highest risk associated with fully refractory disease. The markedly unfavorable prognostic value of primary unresponsive disease was consistently observed in all subtypes, including low-grade lymphoma. Thus, achievement of persistent CR or PR at the initial treatment is the most important prognostic factor in the management of any lymphoma patient. This finding has several implications for any future investigation concerning novel drugs or biosimilar drugs, such as their possible inclusion in first-line treatment for lymphoma patients. [40].
